# An interaction map for HTLV-1 Tax and PDZ-containing proteins

**DOI:** 10.1186/1742-4690-11-S1-P101

**Published:** 2014-01-07

**Authors:** Karim Blibek, Xavier Rambout, Jérôme Beaufays, Laurence Lins, Franck Dequiedt, Jean-Claude Twizere

**Affiliations:** 1 National Fund for Scientific Research, Interdisciplinary Cluster for Applied Genoproteomics (GIGA), University of Liège, Liège, Belgium; 2 National Fund for Scientific Research, Gembloux Agro-Bio Tech, Center of Numerical Molecular Biophysics, University of Liège, Gembloux, Belgium

## 

Human T-cell leukemia virus type 1 (HTLV-1) retrovirus encodes for the Tax protein, which has a transforming capacity in vitro. Tax contains at its C-terminus a binding motif for PDZ domain-containing proteins (PSD95-DLG1-ZO1). It has been shown that the C-terminal motif of Tax is involved in Tax oncogenic capacity. Ten different PDZ domain-containing proteins have been reported to interact with Tax, but the specificity of Tax-human PDZome interactions has not been investigated. The objective of this study is to obtain a comprehensive interactome map for Tax and the human PDZome and to determine a global role of Tax-PDZ interactions in HTLV-1 biology. By using different protein-protein interaction methods we have generated a Tax/human PDZome interaction map. We then performed a clustering analysis to define biological functions associated with Tax/PDZ interactions. PDZ Proteins involved in cell shape, cytoskeleton organization and membrane polarization and traffic were overrepresented, and suggest that Tax/PDZ interactions may be involved in Tax-mediated stimulation of T cell activation pathways.

